# Blockade of Wnt-1 signaling leads to anti-tumor effects in hepatocellular carcinoma cells

**DOI:** 10.1186/1476-4598-8-76

**Published:** 2009-09-24

**Authors:** Wei Wei, Mei-Sze Chua, Susan Grepper, Samuel K So

**Affiliations:** 1 Asian Liver Center, Department of Surgery, Stanford University School of Medicine, Stanford, CA 94305, USA; 2 CellzDirect/Invitrogen, 4301 Emperor Blvd, Durham, NC 27703, USA

## Abstract

**Background:**

Hepatocellular carcinoma (HCC) is an aggressive cancer, and is the third leading cause of cancer death worldwide. Standard therapy is ineffective partly because HCC is intrinsically resistant to conventional chemotherapy. Its poor prognosis and limited treatment options make it critical to develop novel and selective chemotherapeutic agents. Since the Wnt/β-catenin pathway is essential in HCC carcinogenesis, we studied the inhibition of Wnt-1-mediated signaling as a potential molecular target in HCC.

**Results:**

We demonstrated that Wnt-1 is highly expressed in human hepatoma cell lines and a subgroup of human HCC tissues compared to paired adjacent non-tumor tissues. An anti-Wnt-1 antibody dose-dependently decreased viability and proliferation of Huh7 and Hep40 cells over-expressing Wnt-1 and harboring wild type β-catenin, but did not affect normal hepatocytes with undetectable Wnt-1 expression. Apoptosis was also observed in Huh7 and Hep40 cells after treatment with anti-Wnt-1 antibody. In these two cell lines, the anti-Wnt-1 antibody decreased β-catenin/Tcf4 transcriptional activities, which were associated with down-regulation of the endogenous β-catenin/Tcf4 target genes c-Myc, cyclin D1, and survivin. Intratumoral injection of anti-Wnt-1 antibody suppressed *in vivo *tumor growth in a Huh7 xenograft model, which was also associated with apoptosis and reduced c-Myc, cyclin D1, and survivin expressions.

**Conclusion:**

Our results suggest that Wnt-1 is a survival factor for HCC cells, and that the blockade of Wnt-1-mediated signaling may offer a potential pathway-specific therapeutic strategy for the treatment of a subgroup of HCC that over-expresses Wnt-1.

## Background

Hepatocellular carcinoma (HCC) is the primary form of human adult liver cancer. It is the fifth most common cancer worldwide, with about one million new cases diagnosed annually, and almost an equal number of deaths. It is predominant in China, most parts of South East Asia, and South Africa, where hepatitis B virus (HBV) infection is endemic [1]. The last decade has seen no major advances in the treatment of HCC. Approximately 10-25% of HCC patients are candidates for surgical resection and liver transplantation; the majority of patients have limited treatment options due to the lack of effective chemotherapy against this intrinsically resistant tumor [2-4]. New pharmacological interventions that offer even modest improvements in efficacy and disease outcome are eagerly sought.

The Wnt/β-catenin pathway plays an important role in embryogenesis and carcinogenesis [5,6]. Secreted proteins of the Wnt family bind to specific Frizzled (FZD) receptors on the surface of target cells to activate distinct intracellular pathways, resulting in the accumulation and nuclear localization of the β-catenin protein. Nuclear β-catenin binds to T-cell factor 4 (Tcf4) to drive activation of specific target genes including cyclin D1, c-Myc, and survivin, which have been characterized to be critical for cancer development [7-9]. Clinical studies have reported that abnormal activation of Wnt/β-catenin pathway is frequently involved in hepatocarcinogenesis. About 33-67% of HCC tissues show accumulation of β-catenin in the cytoplasm and nucleus, whereas no accumulation was observed in the corresponding normal tissues [10,11]. In addition, FZD7, a receptor for Wnt ligands, was reported to be involved in HCC development and progression [12,13].

The Wnt-1 ligand has been reported to be abnormally expressed in a variety of human cancers including HCC [14,15]. In HCC, proteomics results suggested that enhanced Wnt-1 expression associated with NF-kB might be an important mechanism underlying hepatocarcinogenesis [16]. Moreover, transgenic mice model suggested that high expression of Wnt-1 could be the major cause for nuclear accumulation of β-catenin, which subsequently contributes to c-myc/E2F1-driven hepatocarcinogenesis [17]. Elevated levels of tumor Wnt-1 protein in HBV- and hepatitis C virus (HCV)-related HCC has recently been shown to be a prognostic indicator of HCC recurrence after surgical resection [18]. Because of the functional importance of Wnt-1 in HCC development and progression, we investigated the anti-tumor effects of blocking Wnt-1 mediated signaling through the Wnt/β-catenin pathway in human HCC. By using a polyclonal anti-Wnt-1 antibody, we studied the effects of Wnt-1 blockade on HCC cell growth *in vitro *and *in vivo*, and the effects on Wnt/β-catenin mediated transcriptional activity in HCC cells.

## Results

### Over-expression of Wnt-1 protein in HCC tissue specimens and cell lines

**Figure 1:**
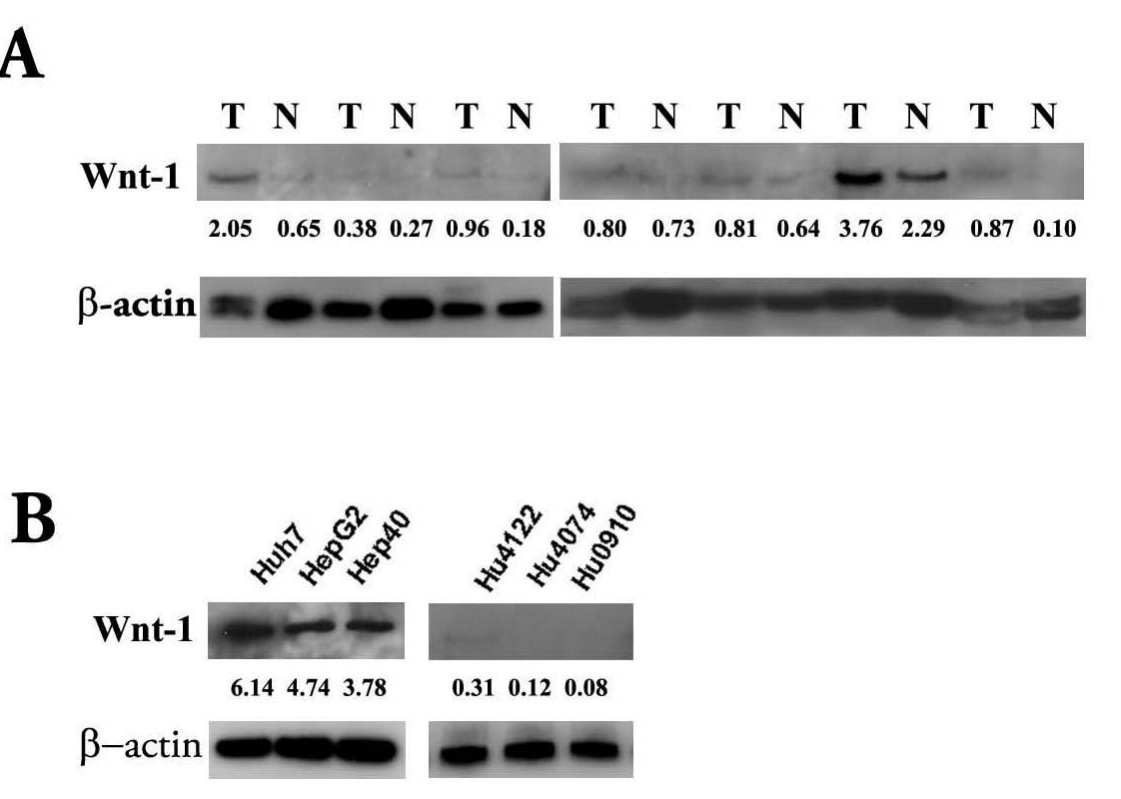
**Expression of Wnt-1 protein in human HCC tumors and cell lines**. A). Western blot detection of Wnt-1 expression in human HCC tumor tissues (T) and the corresponding adjacent non-tumor liver tissues (N). B). Western blot detection of Wnt-1 expression in HCC cell lines (HepG2, Huh7, and Hep40), and normal hepatocytes from three donors (Hu0910, Hu4122, Hu4074). The immunoblots were quantified by densitometry and the intensities normalized with those of β-actin and given beneath each band.

To confirm the expression of Wnt-1 protein in HCC, we used the anti-Wnt-1 antibody to detect its expression in seven pairs of HCC tissues and their corresponding adjacent non-tumor tissues. These tissues were obtained with informed consent from seven HCC patients undergoing surgical resection at Stanford Hospital. Expression of Wnt-1 in HCC tissues was at least 1.5 fold greater than in paired non-tumor tissues in four out of the seven tissue pairs (Fig. 1A). Despite the small sample size, our data closely reflect that reported recently by Lee *et al *[18], who observed that 26 of 63 HCC patients had tumor/non-tumor Wnt-1 expression ratio of ≥ 1.5, whereas 37 of 63 had a ratio of <1.5. Wnt-1 protein expression was in general higher in human HCC cell lines (Huh7, Hep40, and HepG2), but was undetectable in normal hepatocytes cultured from three different donors (Hu4122, Hu4074, Hu0910) (Fig. 1B). In general, our observations corroborate with published reports that Wnt-1 is upregulated in HBV- and HCV-related HCC tissues and cell lines [16,19].

### Anti-Wnt-1 antibody decreases cell proliferation and induces apoptosis in HCC cell lines

**Figure 2:**
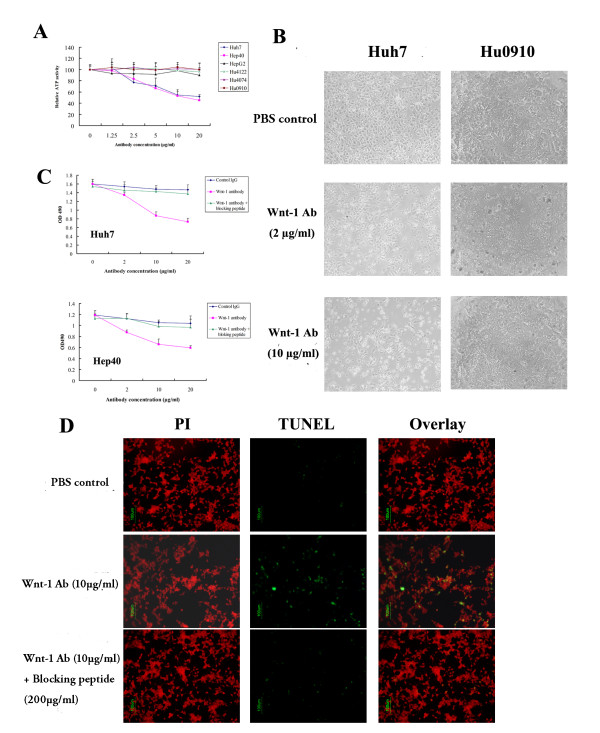
***In vitro *anti-proliferative and apoptotic effects of anti-Wnt-1 antibody on human HCC cell lines**. A). Cell viability assays based on cellular ATP content were used to determine the effect of anti-Wnt-1 antibody on three human HCC cell lines and normal hepatocytes from three donors following 72 hr of antibody treatment. Relative ATP activity is proportional to the number of viable cells. Three independent experiments were done, each in triplicates. B). Phase-contrast microscopic examination of the effect of different concentrations of anti-Wnt-1 antibody on HCC cell line Huh7 and normal hepatocytes Hu0910. C). Pre-treatment with Wnt-1 specific blocking peptide abolished the anti-proliferative effect of anti-Wnt-1 antibody in Huh7 and Hep40 cells. Results are presented as mean ± SD (error bars). D). Anti-Wnt-1 antibody induced apoptosis in Huh7 cells. Huh7 cells were treated with anti-Wnt-1 antibody (10 μg/ml) with or without blocking peptide pre-treatment. After 72 hr incubation, cells were washed, fixed, and stained with TUNEL and PI as described under Materials and Methods to detect for apoptotic cells. Fluorescence labeling was visualized and photographed at 100× magnification.

The over-expression of Wnt-1 in HCC implies that Wnt-1 may be involved in hepatocellular carcinogenesis, and may be critical for the growth of HCC cells. We therefore further studied its potential as a therapeutic target for HCC treatment. After 72 hr exposure, the anti-Wnt-1 antibody dose-dependently decreased cell viability in Huh7 and Hep40 cells, but not in HepG2 cells and normal hepatocytes from three different donors (Fig. 2A and 2B). The lack of activity against HepG2 cells may be due to the presence of truncated β-catenin (with loss of the GSK-3β regulatory site) in these cells, which therefore escapes regulation by upstream events [20]. As a specificity control, we examined the effect on cell proliferation of overnight pre-incubation of the anti-Wnt-1 antibody with its specific blocking peptide (at 20-fold concentration over the antibody). The anti-proliferative effect of anti-Wnt-1 antibody on Huh7 and Hep40 cells were blocked by pre-treatment with the blocking peptide (Fig. 2C), suggesting that the anti-Wnt-1 antibody can bind specifically to the native form of the Wnt-1 protein in these cells, and that the observed anti-proliferative effect is mediated by inhibition of Wnt-1 protein functions by the anti-Wnt-1 antibody.

Using the TUNEL assay, induction of apoptosis was observed at an effective concentration of anti-Wnt-1 antibody in Huh7 (Fig. 2D) and Hep40 cells (data not shown). Most of the dead cells were stained as apoptotic cells when treated with 10 μg/ml of the anti-Wnt-1 antibody for 72 hrs (Fig. 2D). Overnight pretreatment with the Wnt-1 specific blocking peptide (20-fold over antibody) markedly reduced the number of apoptotic cells after treatment with anti-Wnt-1 antibody (Fig. 2D). Our data suggest that cell apoptosis was specifically induced by the anti-Wnt-1 antibody in Huh7 and Hep40 cells.

### Anti-Wnt-1 antibody inhibits Wnt/β-catenin signaling and suppresses the expression of downstream oncoproteins

**Figure 3:**
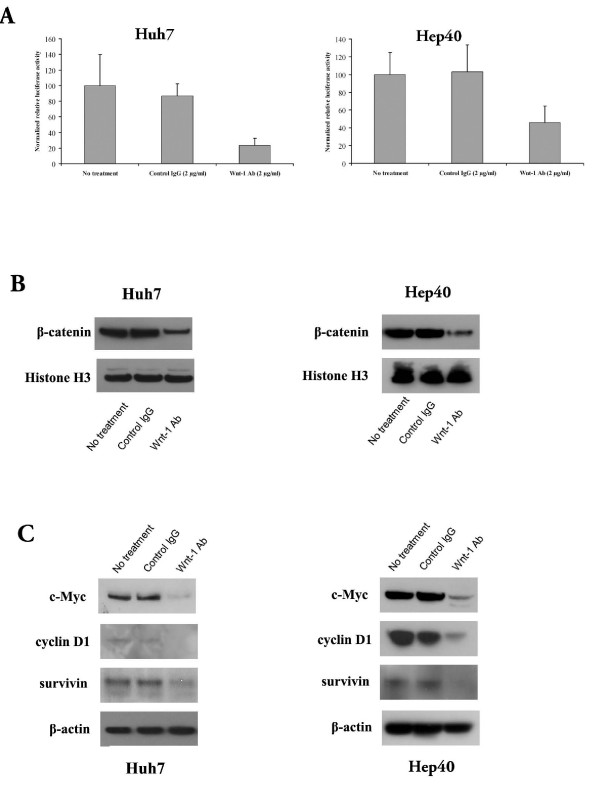
**Anti-Wnt-1 antibody inhibited β-catenin/Tcf4 transcriptional activity**. A). Tcf4 reporter assay of Tcf-dependent transcriptional activity in Huh7 and Hep40 cell lines. Huh7 and Hep40 cells were co-transfected with plasmid encoding β-galactosidase (a control for transfection efficiency) and either the pTOPFLASH or pFOPFLASH reporters. Cells were incubated with anti-Wnt-1 antibody or control IgG (2 μg/ml) and harvested after 48 hr to measure luciferase and β-galactosidase activities. Reporter gene activation is expressed in terms of relative light units (RLU) detected in pTOPFLASH or pFOPFLASH transfected cells and normalized for β-galactosidase activity. The results are expressed as mean ± SD (error bars). Experiments were performed in triplicates; P < 0.05. B). Anti-Wnt-1 antibody decreased nuclear β-catenin accumulation in Huh7 and Hep40 cells. Histone 3 was used as the loading control. C). The effect of anti-Wnt-1 antibody on the expression of β-catenin/Tcf4 target genes c-Myc, cyclin D1, and survivin. Huh7 and Hep40 cells were incubated for 48 hr with anti-Wnt-1 antibody (2 μg/ml) and c-Myc, cyclin D1, survivin and β-actin (loading control) levels were determined by Western blotting using specific antibodies.

To determine if the effects of anti-Wnt-1 antibody on Huh7 and Hep40 cells were related to inhibition of Wnt/β-catenin signaling, we used the Tcf4 transcriptional reporter (TOP/FOP FLASH) assay to detect β-catenin/Tcf4 transcriptional activity in these cell lines after 48 hr treatment with the antibody (2 μg/ml). The anti-Wnt-1 antibody dose-dependently decreased β-catenin/Tcf4 transcriptional activity (Fig. 3A), and also reduced the accumulation of β-catenin in the nuclei of Huh7 and Hep40 cells (Fig. 3B). No effects on β-catenin/Tcf4 transcriptional activity or β-catenin accumulation were observed in HepG2 cells (data not shown). Control IgG had no effect on transcriptional activity or nuclear β-catenin levels. Consistent with these observations, the endogenous levels of β-catenin/Tcf4 regulated proteins (c-Myc, cyclin D1, and survivin) were reduced after 48 hr treatment with the anti-Wnt-1 antibody (2 μg/ml), but not after treatment with control IgG (Fig. 3C). These three proteins are commonly known to be over-expressed in HCC tissues [7-9], and our results indicate that their expression is regulated by Wnt-1-mediated β-catenin/Tcf4 signaling.

### Anti-Wnt-1 antibody inhibits growth of HCC xenografts in nude mice

**Figure 4:**
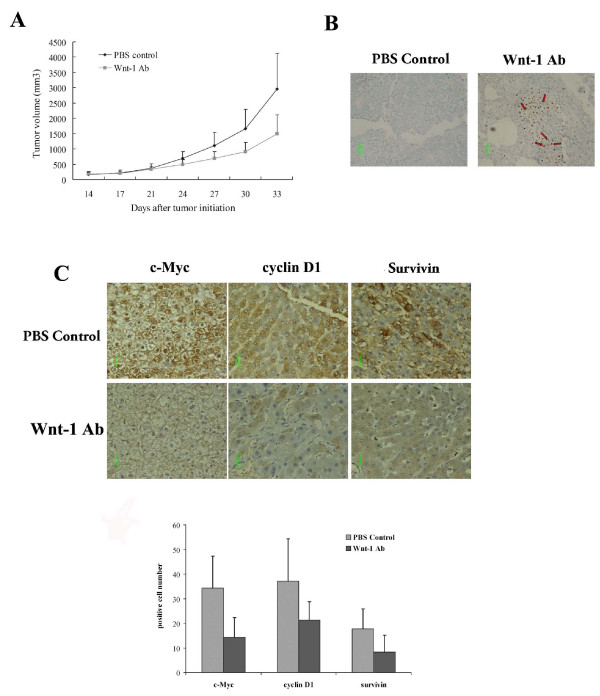
**Anti-Wnt-1 antibody suppressed tumor xenograft growth *in vivo***. A). Nude mice bearing Huh7 tumor xenografts were treated with anti-Wnt-1 antibody (dose: 50 μg/kg; once a week), or with PBS as a control (n = 5 in each group). Significant differences in the tumor volumes between anti-Wnt-1 antibody-treated and PBS control groups were observed at the beginning of the third week after initiation of treatment (P < 0.05). B). TUNEL staining of the xenograft specimens removed from controls and mice treated with anti-Wnt-1 antibody (200× magnification). Red arrows indicate the positively stained apoptotic cells. C). Xenograft specimens removed from controls and mice treated with anti-Wnt-1 antibody were immunostained to detect expression of c-Myc, cyclin D1, and survivin (400× magnification). The number of positively stained cells were counted from three randomly selected areas and the values represent mean ± SD (error bars).

We next studied the *in vivo *effects of the anti-Wnt-1 antibody on tumor growth in a HCC xenograft model in nude mice. Huh7 cells were injected subcutaneously into nude mice to initiate tumor formation. When established xenografts were palpable (approximately after 14 days), mice were intratumorally injected with the anti-Wnt-1 antibody (50 μg/kg), and PBS as control (n = 5 in each group) once weekly. Compared with PBS control, the anti-Wnt-1 antibody effectively inhibited tumor growth *in vivo *(ANOVA, P < 0.05, compared with PBS control at the beginning of the third week) (Fig. 4A). Tumor tissues were harvested after sacrificing the mice at the end of the treatment period, and were analyzed *via *TUNEL staining. Apoptotic cells were detected in tumors treated with the anti-Wnt-1 antibody, but not in the control group (Fig. 4B). When immunostained with anti-c-Myc, cyclin D1, and survivin antibodies, tumor tissues that had been treated with anti-Wnt-1 antibody showed reduced c-Myc, cyclin D1, and survivin expressions (Fig. 4C and 4D). These results are consistent with our *in vitro *observations that the antibody inhibited β-catenin/Tcf4 mediated transcriptional activity.

## Discussion

The Wnt gene family encodes at least 19 secreted factors involved in cell growth, differentiation, embryogenesis and oncogenesis [21]. The expression of the Wnt-1 ligand in mammalian cells results in the accumulation of steady-state levels of cytosolic and nuclear β-catenin [22], which contributes to the development of many types of cancers [6]. Recently, several Wnt proteins, including Wnt-1, have been shown to be over-expressed in HCC tumor compared with the corresponding non-tumor tissue from hepatitis B and C-infected patients [16]. Moreover, *in vitro *models have been used to study how the over-expression of Wnt-1 is involved in HCV-induced Huh-7 cell proliferation and HBV X protein-induced β-catenin/Tcf4 transcription activation [19,23]. Consistent with these recent findings, we have shown that Wnt-1 protein is over-expressed in HCC tissues and cell lines, but not in adjacent non-tumor liver tissues or cultured normal hepatocytes. Our *in vitro *and *in vivo *data further support the use of an anti-Wnt-1 antibody as a new treatment option for HCC, since it blocks Wnt-1 mediated β-catenin/Tcf4 transcription, which in turn leads to reduced proliferation, enhanced apoptosis, and down-regulation of important downstream target oncoproteins in HCC.

Wnt-1-mediated β-catenin/Tcf4 transcription is an important cellular survival pathway that promotes cell proliferation, cell cycle progression, and cellular transformation [24]. Because Wnt proteins are indispensible for cancer cell growth and survival, we studied the potential anti-tumor effect of anti-Wnt-1 antibody in HCC cells. Compared to the control goat IgG, anti-Wnt-1 antibody decreased proliferation and induced apoptosis in Huh7 and Hep40 cell lines over-expressing Wnt-1 protein (and with wild type β-catenin). These effects were blocked by pre-treatment with the Wnt-1 blocking peptide, demonstrating that they were mediated by specific binding of the anti-Wnt-1 antibody to the native form of Wnt-1 protein in these cells. However, the anti-Wnt-1 antibody had no effect on HepG2 cells (with high expression of Wnt-1 protein, but harbors both wild type and truncated β-catenin) [25], and normal hepatocytes from three different donors (with undetectable Wnt-1 expression). Truncation of β-catenin in HepG2 cells is caused by a large gene deletion (loss of amino acids 20-140) involving loss of the GSK-3β regulatory site, which leads to failure of the protein to be phosphorylated by GSK3β. Thus, β-catenin constitutively accumulates in the nucleus of HepG2 cells and may escape regulation by the anti-Wnt-1 antibody acting upstream. Similar results were observed by Shih *et al*. [20], who found that the restoration of secreted frizzled-related proteins (SFRPs; extracellular signaling glycoproteins that antagonize Wnt signaling) attenuated Wnt signaling in Huh6 cells (with a β-catenin point mutation), but not in HepG2 cells with truncated β-catenin. However, as the incidence of similar β-catenin truncation is low in human HCC (only 1 out of 26 HCC patients had β-catenin truncation) [25], the anti-Wnt-1 antibody has potential wide applicability in the majority of HCC patients.

Activation of β-catenin/Tcf4 transcription by Wnt-1 has been reported to be responsible for Wnt-1-mediated cell survival [24]. Longo *et al*. [26] also demonstrated that Wnt-1 caused 3T3-L1 cells to resist apoptosis in response to serum deprivation through inhibition of GSK-3β activity and stabilization of β-catenin. Conversely, blockade of Wnt-1 signaling using Wnt-1 antibodies have been reported to induce apoptosis in a variety of human cancer cell lines, including non small cell lung cancer, breast cancer, colorectal cancer mesothelioma, and sarcoma [27,28]. Treatment with antibodies against Wnt-1 and Wnt-10b also inhibited proliferation and induced apoptosis in head and neck squamous cell carcinoma (HNSCC) cells [29]. Consistently, we observed that anti-Wnt-1 antibody inhibited β-catenin/Tcf4 transcriptional activity as measured using the TOPFLASH and FOPFLASH reporter assay, and also led to accumulation of β-catenin in the nuclei of Huh7 and Hep40 cells. This implies that anti-Wnt-1 antibody acts *via *the canonical Wnt signaling cascade to induce apoptosis in both cell lines.

The canonical Wnt signaling pathway, *via *the β-catenin/Tcf4 complex, regulates the expression of multiple oncoproteins, including c-Myc, cyclin D1, and survivin in HCC [7-9]. In the Huh7 and Hep40 cells *in vitro*, anti-Wnt-1 antibody markedly reduced the expression of c-Myc, cyclin D1, and survivin, consistent with its ability to inhibit β-catenin/Tcf4 transcriptional activity. One of the most common oncoproteins associated with the pathogenesis of HCC is c-Myc [8]; its elevated expression in mice models was found to initiate and promote liver tumor growth [30,31]. The deletion of c-Myc alone in a mouse liver tumor model efficiently suppressed tumor growth *in vivo *[32]. Cyclin D1 was reported to be significantly elevated in HCC compared with surrounding cirrhotic tissues [9]. In HCC cells, cyclin D1-related kinase activity was accompanied by up-regulation of Cdk4 activity, and phosphorylated and activated pRB, which promote G1 to S phase transition [9]. Additionally, the over-expression of survivin promotes proliferation in HCC by initiating cell cycle entry (a decrease in the G0/G1 phase and an increase in the S phase) [7]. Its inhibition led to cell cycle arrest, an effect which can augment the sensitivity of HCC tumor cells to cytotoxic drugs [33]. These three oncoproteins (c-Myc, cyclin D1, and survivin) have important functional roles in the development of HCC, and the ability to regulate them simultaneously offers a powerful way to intervene with HCC progression.

## Conclusion

In conclusion, our data suggest that targeting Wnt-1 mediated Wnt/β-catenin signaling (which is frequently activated in HCC) may be a feasible and novel therapeutic option. The specific and effective blockade of this signaling pathway using an anti-Wnt-1 antibody inhibited HCC cell proliferation and induced apoptosis *in vitro*. Correspondingly, suppression of HCC tumor xenograft growth and apoptosis were also observed *in vivo*. The ability of the anti-Wnt-1 antibody to down-regulate the expression of downstream targets of Wnt/β-catenin signaling, including c-Myc, cyclin D1, and survivin, suggest that a broad spectrum of cellular mechanisms can be triggered in concert to halt HCC tumor growth. Lastly, since Wnt-1 is often deregulated in HCC with underlying HBV or HCV (the main risks factors of HCC), we propose that interference with Wnt-1 mediated signaling offers a potent and selective therapeutic strategy for the clinical management of a substantial proportion of HCC patients with Wnt-1 over-expression.

## Material and methods

### Tissue samples and cell lines

Paired (HCC and adjacent non-tumor liver) tissues were obtained from seven patients who underwent liver resection for HCC at Stanford Hospital, Stanford University, California. This study was approved by the Institutional Review Board for the use of human subjects in medical research, and informed consent was obtained from patients prior to liver resection. Ages ranged from 44 to 73 years. All patients were men with HBV-related HCC.

Human hepatoma cell lines, HepG2, Hep40, and Huh7 were maintained in Dulbecco's Modified Eagle's Medium (DMEM) supplemented with 10% fetal bovine serum (FBS), 100 μg/mL penicillin and 100 μg/mL streptomycin. All media and supplements were from Invitrogen (Carlsbad, CA). Cells were maintained at 37°C in a humidified atmosphere with 5% CO_2_.

### Anti-Wnt-1 antibody and blocking peptide

The anti-Wnt-1 goat polyclonal antibody, the corresponding control goat IgG, and the Wnt-1 specific blocking peptide were from Santa Cruz Biotechnology (Santa Cruz, CA). Antibodies were concentrated using Microcon-30 ultrafugation devices (Millipore Corporation, Bedford, MA) before being added to cells. The blocking peptide was purified by dialysis using a Slide-A-Lyzer Dialysis Cassette with a 2,000 MWCO (Promega, Madison, WI). For blocking experiments, blocking peptide was pre-incubated with cells overnight, at 20-fold concentration over the anti-Wnt-1 antibody.

### Primary culture of hepatocytes

Cryopreserved human hepatocytes, collagen hand-coated 96-well plates, CHRM Thawing Medium, Cell Plating Medium, and Cell Maintenance Medium were received from CellzDirect/Invitrogen (Durham, NC). Characteristics of the three hepatocyte lots are shown in Table 1. Cryopreserved human hepatocytes were thawed based upon CellzDirect's standard method: hepatocytes were thawed at 37°C, then poured into pre-warmed 37°C CHRM Thawing Medium at a ratio of one vial (approximately 5 million cells)/50 ml in a conical tube. The cells were then centrifuged at 100g for 10 min and resuspended to 0.75 × 10^6 ^cells/mL in Plating Medium. Cell viability was determined by trypan blue exclusion. Hepatocytes were then plated in collagen-coated 96-well plates at a density of 3 × 10^4 ^cells/well in a volume of 100 μl/well. After 5 h of incubation at 37°C, Plating Media was replaced with serum-free Maintenance Media at 100 μl/well, and incubated overnight for cytotoxicity assay as described below.

**Table 1:** Characteristics of tested normal hepatocytes

**Lot**	**Age (y)**	**Gender**	**Race**	**Cell viability**
Hu4122	19	Male	Caucasian	92%

Hu0910	56	Female	Caucasian	96%

Hu4074	47	Male	Caucasian	87%

### Cell viability and proliferation assays

Hepatoma cells were seeded in 96-well plates at 3 × 10^3 ^cells/well, and incubated overnight at 37°C prior to addition of anti-Wnt-1 antibody. Anti-Wnt-1 antibody and control IgG were added at desired final concentrations (range from 0-20 μg/ml), and further incubated for 72 h before cell viability and proliferation were assessed using CellTiter-Glo Luminescent Cell Viability Assay (Promega, Madison, WI) or .CellTiter 96^® ^AQueous One Solution Cell Proliferation Assay (Promega, Madison, WI) respectively, according to the manufacturer's instructions. Briefly, for the .CellTiter-Glo assay, the assay regents (a combination of detergent and luciferase-based enzyme) were directly added to cultured cells, resulting in cell lysis and the production of a bioluminescent signal proportional to the amount of ATP present. Luciferase activity was measured on a luminometer (Berthold LB-96V) and values were normalized to the ATP activity and compared with their respective PBS control value, which was set at 100. Three independent experiments were done, each in triplicates. For proliferation assay, optical density (OD) was read at 490 nm using a SAFIRE microplate reader (TECAN, Research Triangle Park). The background value (mean OD values from wells with only the AQueous One Solution) was subtracted from all data. Three independent experiments were done, each in triplicates.

### Luciferase reporter gene assay

β-catenin/Tcf4 transcriptional reporter gene assays were performed using TCF/Luc reporter constructs, wild type pTOPFLASH, and mutant pFOPFLASH, which were generously provided by B Vogelstein (John Hopkins Oncology Center, Baltimore, MD, USA) [34]. Cells were seeded at 3 × 10^4 ^cells/well into 24-well plates and incubated for 24 hr prior to transfection with wild type pTOPFLASH or mutant pFOPFLASH (0.7 μg) using Lipofectamine 2000 (Invitrogen, Carslbad, CA) according to the manufacturer's instructions. The β-galactosidase (β-gal) expression vector (0.1 μg) was added to each transfection system to normalize the transfection efficiency. After 4 hr, medium containing transfection regent was replaced with new culture medium containing the anti-Wnt-1 antibody or control IgG (2 μg/ml). After 48 hr, cells were lysed in 100 μl of lysis buffer, and 20 μl aliquots were assayed for luciferase activity using the Promega Luciferase assay system or for β-gal activity using the Promega β-gal assay system. Relative light units (RLU) were measured and normalized for transfection efficiency using β-gal activity. Final RLU representing Tcf4 transcriptional activity were calculated by subtracting normalized levels obtained with pFOPFLASH from those obtained with pTOPFLASH.

### Apoptosis Analysis

TUNEL (Terminal dUTP nick-end labeling) assays (Promega, Madison, WI) were performed according to the manufacturer's protocol. Briefly, Huh7 or Hep40 cells were seeded in 8-chamber BD tissue culture slides (BD Bioscience Labware, Bedmord, MA) at 10% confluency. Control IgG or anti-Wnt-1 antibody was added to the medium at final concentration of 10 μg/ml each. After 72 hr incubation, cells were washed twice with PBS, and then fixed in 4% paraformaldehyde for 25 min. Fixed cells were washed twice in PBS with 0.1% Triton X-100, and then incubated with TUNEL reaction mixture for 60 min at 37°C. After washing with 2xSSC, slides were immersed in PBS with 1 μg/ml Propidium Iodide (PI) for 5 min in the dark and then washed with PBS. Fluorescence labeling was visualized and photographed (100× magnification) with a fluorescence microscope (Nikon Eclipse 80i, Nikon Corporation, Tokyo, Japan) and with a digital camera (Nikon DXM1200f, Nikon Corporation, Tokyo, Japan). For TUNEL staining of the tumor xenografts, 4-μm tissue sections of tumor xenografts from *in vivo *experiments were stained using the ApopTag Peroxidase in Situ Oligo Ligation Apoptosis Detection Kit (Chemicon International, Temecula, CA) according to the manufacturer's protocol.

### Western blotting and antibodies

Huh7 or Hep40 cells were seeded at 50% confluency in 6-well plates and incubated at 37°C overnight. Cells were then treated with PBS, control IgG, or anti-Wnt-1 antibody (2 μg/ml) for 48 hr. Cell monolayers were washed twice with PBS and then lysed in RIPA extraction buffer. For nuclear β-catenin immunodetection, nuclear extracts were prepared with a NE-PER Nuclear and Cytoplasmic Extraction Kit (Pierce, Rockford, IL). Equal amounts of protein (20 μg) were resolved by SDS-PAGE and Western blots were performed by using the primary antibodies to c-Myc (1:500, Cat. 551101, BD Pharmingen, San Diego, CA), cyclin D1 (1:1000, Cat. ab6152, Abcam, Cambridge, MA),, survivin (1:1000, Cat. NB500-201H, Novas Biologicals, Littleton, CO), β-catenin (1:500; Cat. SC-7963, Santa Cruz Biotechnology, Santa Cruz, CA), Histone H3 (1:10000, Cat. Ab21054, Abcam, Cambridge, MA) or β-actin (1:10000, Cat.A3854, Sigma-Aldrich, MO). Secondary antibodies (anti-mouse, Cat.SC-2005, and anti-rabbit, Cat.SC-2004) conjugated with horseradish peroxidase were obtained from Santa Cruz Biotechnology (Santa Cruz, CA). Western blots were analyzed by ImageJ software, and signal intensities normalized to β-actin.

### Xenografts in Nude Mice

Nude mice (ATHYMIC NU/NU; Harlan Sprague-Dawley, Indianapolis, IN) at age 4-6 weeks with a body weight of 18 to 25 g were used for the experiments. Mice were injected subcutaneously at the dorsal region with 5 × 10^6^/150 μl viable Huh7 cells. After two weeks, when tumors reached approximately 0.4-0.5 cm in diameter, mice were randomized into groups (n = 5) to be intratumorally injected with 100 μl of PBS or anti-Wnt-1 antibody at the dose of 50 μg/kg once a week. Tumor size was measured with digital calipers every three days and was calculated using the formula π/6 × larger diameter × [smaller diameter]^2^. Mice were sacrificed at the end of the treatment period, and xenografts harvested.

### Immunohistochemical Analysis

Immunoperoxidase stain of tumor xenografts was performed on acetone-fixed 4-μm tissue sections. Briefly, sections were incubated with monoclonal mouse anti-human c-Myc (1:200, Cat. 551101, BD Pharmingen, San Diego, CA), cyclin D1 (1:250, Cat. ab6152, Abcam, Cambridge, MA), or survivin (1:500, Cat. NB500-201H, Novas Biologicals, Littleton, CO) and then washed with PBS. Subsequent procedures were performed using Dakocytomation Envision System-HRP mouse system (DakoCytomation Inc, CA, USA) according to the manufacturer's protocol. We observed c-Myc staining in the cytoplasm and nuclei, and cyclin D1 and survivin staining in the cyptoplasm, consistent with other reports in HCC [35-37]. Therefore, for quantification, 100 cells at 3 randomly selected areas were assessed, and the number of cells that stained positively for c-Myc, cyclin D1, or survivin were counted.

### Statistical analysis

Statistical analysis was performed by one-way ANOVA and independent-sample T-test using the computer SPSS software. In all assays, the probability value (P) of < 0.05 was considered statistically significant.

## Abbreviations

HCC: Hepatocellular carcinoma; HBV: hepatitis B virus; Tcf4: T-cell factor 4; TUNEL: Terminal dUTP nick-end labeling; PI: propidium iodide

## Competing interests

The authors declare that they have no competing interests.

## Authors' contributions

WW contributed to the major part of experimental work, interpreted the results, performed the statistics and drafted the manuscript. MC conceived the study, participated in its design and data analysis, and contributed with scientific discussion and manuscript preparation. SG contributed the normal hepatocytes and provided training and advice on culturing the hepatocytes. SKS is the principal investigator, responsible for conception of the project, designing the experiments, and approving the final manuscript. All authors read and approved the final manuscript.
